# Statistical tests for intra-tumour clonal co-occurrence and exclusivity

**DOI:** 10.1371/journal.pcbi.1009036

**Published:** 2021-12-15

**Authors:** Jack Kuipers, Ariane L. Moore, Katharina Jahn, Peter Schraml, Feng Wang, Kiyomi Morita, P. Andrew Futreal, Koichi Takahashi, Christian Beisel, Holger Moch, Niko Beerenwinkel

**Affiliations:** 1 Department of Biosystems Science and Engineering, ETH Zurich, Basel, Switzerland; 2 SIB Swiss Institute of Bioinformatics, Basel, Switzerland; 3 Department of Pathology and Molecular Pathology, University and University Hospital Zurich, Zurich, Switzerland; 4 Department of Genomic Medicine, The University of Texas MD Anderson Cancer Center, Houston, Texas, United States of America; 5 Department of Leukemia, The University of Texas MD Anderson Cancer Center, Houston, Texas, United States of America; National Center for Biotechnology Information (NCBI), UNITED STATES

## Abstract

Tumour progression is an evolutionary process in which different clones evolve over time, leading to intra-tumour heterogeneity. Interactions between clones can affect tumour evolution and hence disease progression and treatment outcome. Intra-tumoural pairs of mutations that are overrepresented in a co-occurring or clonally exclusive fashion over a cohort of patient samples may be suggestive of a synergistic effect between the different clones carrying these mutations. We therefore developed a novel statistical testing framework, called GeneAccord, to identify such gene pairs that are altered in distinct subclones of the same tumour. We analysed our framework for calibration and power. By comparing its performance to baseline methods, we demonstrate that to control type I errors, it is essential to account for the evolutionary dependencies among clones. In applying GeneAccord to the single-cell sequencing of a cohort of 123 acute myeloid leukaemia patients, we find 1 clonally co-occurring and 8 clonally exclusive gene pairs. The clonally exclusive pairs mostly involve genes of the key signalling pathways.

## Author summary

Tumours typically display high levels of heterogeneity, not only between different tumours but also within a single one. Intra-tumour heterogeneity results from an evolutionary process, giving rise to different populations of cancer cells known as clones. How clones interact may affect tumour evolution, which in turn determines disease progression and treatment outcome. In practice, we may observe pairs of mutations that co-occur in clones or exclude each other more often than we would expect for a given cohort of patient samples. Exclusive pairs are suggestive that clones carrying one or the other mutation may cooperate in the evolutionary process. Targeting only one of them may then suffice to alter the tumour evolution. Therefore it is critical to have statistical methods which allow us to identify such pairs. GeneAccord is a novel statistical testing framework we developed especially to identify pairs of genes altered in distinct clones of the same tumour. Accounting for the evolutionary dependencies among clones emerged as critical to adequately control testing errors. In a cohort of 123 acute myeloid leukaemia patients, GeneAccord identified one clonally co-occurring and eight clonally exclusive gene pairs. The latter predominantly involved genes of key signalling pathways.

## Introduction

Intra-tumour heterogeneity refers to a diverse set of genetically or phenotypically distinct cell populations that coexist within a tumour [1, 2]. It is the result of mutation, selection, and possibly other evolutionary forces during tumour evolution [3–5]. More diverse tumours support more evolutionary pathways and tend to adapt better to treatment and immune responses. Hence they are more likely to escape these selective pressures and develop therapy resistance or immune escape [6, 7]. Profiling tumours [8] and their heterogeneity therefore has the potential to improve cancer diagnostics and treatment, and the ability to resolve the clonal and subclonal structure of tumours has progressed rapidly with multi-region bulk sequencing [9] and single-cell sequencing [10, 11]. Recently, for acute myeloid leukeamia (AML), high-throughput single-cell panel sequencing has uncovered the clonal diversity across two cohorts of 123 patients [12, 13]. These studies show that AML samples tend to have a relatively small number of clones, and importantly that multiple different mutations in signalling pathway genes often occur in distinct subclones.

Beyond offering more potential evolutionary pathways, intra-tumour heterogeneity provides more than just a bet-hedging strategy since the individual clones may interact with each other to confer communal advantages [14–16]. Interaction processes between clones are distinct from genetic interactions (epistasis), where, for instance, two genes are mutated in the same cell or clone and together lead to an unanticipated change in the phenotype [17]. The different types of ecological interactions among cancer clones [16] include negative interactions, for example when clones compete for nutrients or oxygen, while positive interactions such as commensalism, synergism, and mutualism drive tumour cell proliferation and ultimately favour greater intra-tumour heterogeneity. One example of commensalism, where one clone benefits from another one without providing anything in return, is a clone stimulating blood vessel growth, which also supplies other surrounding cells with nutrients and oxygen [18]. *Ras*-mutated dermal fibroblast cells have been observed to secrete factors that lead to down-regulation of a strong angiogenesis inhibitor in normal cells over 10mm away [19], also suggesting that interactions may not only occur between directly neighbouring clones in solid tumours. Cooperation, including synergism and mutualism, where for example, different clones cross-feed each other resources, was hypothesised to be a driving factor in tumour progression [18] and a wealth of examples of clonal cooperation have been discovered in cancer [20–27]. If a combination of two or more clones is beneficial, these clones will likely co-exist stably over time and not outcompete each other [18, 24]. More formally, cooperation between cancer clones can be modelled and interpreted using evolutionary game theory [28–30].

As cooperation can play a significant evolutionary role in tumour progression, it is important to elucidate its underlying mechanisms. In particular, understanding how to disrupt this process potentially opens up novel treatment strategies to improve personal cancer treatment. In order to investigate how clones co-exist and possibly interact, a systematic screening of subclonal mutation compositions is necessary. From bulk sequencing data, co-occurrence and mutual exclusivity patterns can be detected from the clonal mutation patterns across cohorts of patients [31–37]. This patient-level resolution however does not consider the subclonal structure within each patient overlooking potential subclonal interactions. Intra-tumour co-occurrence and exclusivity patterns may differ substantially from the patient level ones. For example, if two genes are strictly mutually exclusive at the patient level, they never both occur in the same or different clones and cannot have subclonal interactions. On the other hand, if genes are exclusive at the clone level and only appear in different subclones, they can still both occur and produce co-occurrence at the patient level.

Resolving tumours at the subclonal level with multi-region bulk or single-cell sequencing offers a route to address intra-tumour co-occurrence and exclusivity. However it involves another challenge: the genotypes of the subclones are not independent observations. Hence, treating clones like tumour samples in the above-mentioned patient-level analyses would lead to spurious correlations. Instead, we must account for the dependency structure among clones encoded by their phylogenetic relationships. For example, for two mutations in a common lineage, after the first mutation we have clones with the genotype (1, 0) resembling an exclusivity pattern while after both mutations we have the genotype (1, 1) indicating co-occurrence. To avoid treating the dependency structure, previous analyses have, for example, been limited to the common ancestor clone of the mutations to ensure independence, as was the case for the analysis of clonal co-occurrences and exclusivities of ten selected driver events in clear cell renal carcinoma using multi-region bulk sequencing [9].

Here we develop a statistical testing framework, called GeneAccord, which considers the full phylogenetic tree representing the complete evolutionary history of each tumour and the subclonal mutation patterns across the entire tree. To uncover clonal co-occurrence or exclusity, GeneAccord analyses (*i*) the placement of mutations within the phylogenetic trees of the patients exhibiting the mutations, (*ii*) the occurrence of the mutations across the cohort and which patient trees contain the mutations, and (*iii*) the combination of these two signals in a joint test. GeneAccord can additionally take into account the uncertainty in the tree inference by allowing the input of multiple alternative tree topologies per patient. With this input, GeneAccord assesses, in a statistically rigorous way, whether specific subclonal mutation combinations occur at a higher or lower rate than expected by chance over the cohort of patient tumour samples. We evaluate GeneAccord’s power and demonstrate that it correctly controls the type I error rate, unlike baseline alternatives which do not account for the underlying phylogenetic trees. Finally, we illustrate GeneAccord on a cohort of 123 AML patients resolved with single-cell sequencing [12]. We find significant signs of clonal exclusivity, particularly between genes involved in signalling pathways.

## Results

### GeneAccord algorithms

**Fig 1:**
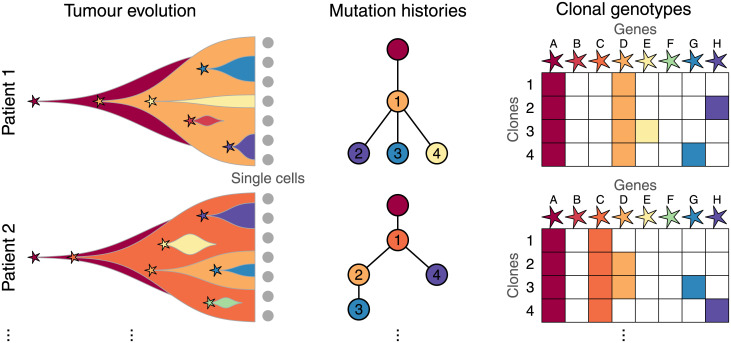
Tumour evolution and clonal exclusivity. During tumour evolution different mutations may arise leading to heterogeneous subclones with distinct genotypes. From single-cell sequencing we may reconstruct the mutational history of each tumour, encoding the ordering of mutations and their phylogenetic relationships. The clones are numbered in each tree and inherit mutations in ancestral clones. For example in patient 1, clone 2 exhibits mutations in genes A, D and H (red, orange, purple) as displayed in the clone-genotype matrix on the right. Clonally exclusive mutations will appear in different branches of the trees and exhibit mutually exclusive patterns in the clone-genotype matrices, as exhibited for example for the two rightmost mutations G and H (blue and purple) in both patients.

In order to systematically analyse subclonal mutation combinations in tumour clones, we developed a statistical framework and implemented it in the R package GeneAccord. The statistical tests can identify pairs of mutated genes or pathways that both occur in the same tumour but in different clonal lineages. For a particular tumour, we define a gene pair as clonally exclusive if there are two clone lineages in the tumour such that one of them possesses mutations in only one of the genes, while the other lineage has mutations only in the other gene. The complement of being clonally exclusive is clonal co-occurrence, where a common clonal lineage exists that contains both mutations. By resolving the evolutionary history of tumours, for example through single-cell sequencing and reconstructing the genotypes of the clones, clonally exclusive gene pairs will display mutual exclusivity (Fig 1). The underlying rationale for searching for clonally exclusive gene pairs is that if two clones co-exist in a tumour and cooperate, for example, by sharing diffusible factors, they may have acquired complementary sets of mutations for mutual benefit.

To search for clonal co-occurrence or exclusivity, we evaluate across a cohort of patient tumours whether the observed patterns of exclusivity occur more or less often than expected by chance alone. Specifically, we developed three tests to uncover signals of clonal co-occurrence or exclusivity: (*i*) a gene pair *placement* test which, for patient trees exhibiting both mutations, compares the locations of the mutations to a null model of random placement within each tumour phylogeny; (*ii*) a gene pair *occurrence* test which compares which patient trees exhibit both mutations to a null model of random occurrence across the patient cohort; and (*iii*) a *combined* test of clonal co-occurrence or exclusivity which evaluates the joint signal from the placement and occurrence tests.

We frame the testing procedures as likelihood ratio tests with a single clonal exclusivity score Δ for each gene pair, which when negative indicates that the gene pair is mutated in different clones more often than expected, and when positive indicates a higher rate of co-occurrence in the same clonal lineage. We developed and implemented the statistical tests in the GeneAccord R package (Methods, https://github.com/cbg-ethz/GeneAccord). After testing gene pairs, multiple testing correction is performed to control the false discovery rate with the Benjamini-Hochberg procedure [38]. If a pair is significantly clonally exclusive, it suggests that this specific clone configuration may confer a selective advantage, possibly through cooperation between the clones.

### Calibration and power of the placement test

**Fig 2:**
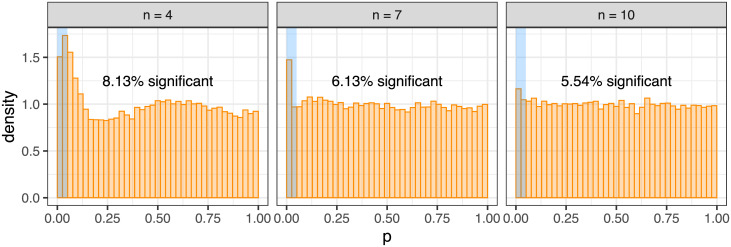
Calibration of the chi-squared approximation for the gene pair placement test. For gene pairs simulated to occur in *n* patient samples, for smaller *n* we observe overly liberal p-values and a lack of calibration with the chi-squared approximation for the gene pair placement test. For larger *n*, the approximation becomes more appropriate.

The statistical test of gene pair placement (Methods) is a likelihood ratio test where the test statistic asymptotically follows a chi-squared distribution. For gene pairs observed in a smaller number *n* of patient samples this asymptotic result can however be poorly calibrated. For example, we simulated data under the null where the background clonal exclusivity rates *r* are sampled from a beta distribution with parameters 2 and 3 which gives a similar mean and variance to the rates computed from the real AML dataset. Using the chi-squared distribution to compute p-values under the null we see an enrichment of lower p-values for smaller *n* (Fig 2). The chi-squared approximation however seems appropriate when the gene pair is observed in enough samples (more than ∼10).

**Fig 3:**
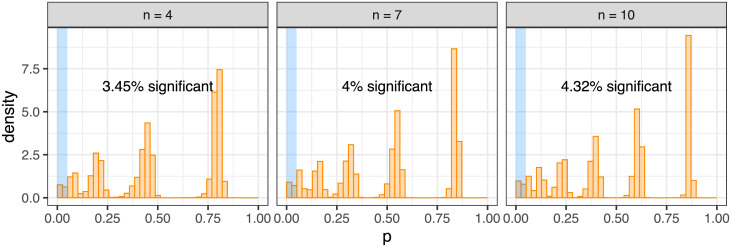
Calibration of the exact gene pair placement test. For the exact test on the simulated data of Fig 2 we observe conservative p-values at lower significance levels, and very pronounced discrete effects for larger values.

We therefore developed an exact version of the placement test (Methods) to ensure calibration. For the same simulated example as before we now observe fewer than 5% of p-values under the null being significant at a 5% threshold (Fig 3). However, since the number of possible outcomes is limited, we also observe very strong discrete effects in the null p-value distribution. For larger *n*, enumerating all possible outcomes for the exact placement test becomes more computationally expensive, while the chi-squared approximation improves. As a default in the R package we therefore switch to the chi-squared approximation for *n* > 12.

**Fig 4:**
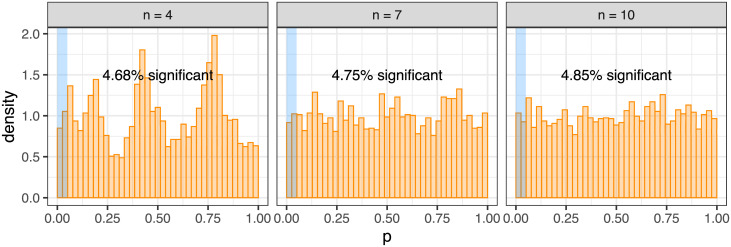
Calibration of the exact gene pair placement test with Monte Carlo smoothing. Smoothing the exact gene pair placement test in Fig 3 by adding noise to the rates, we observe good calibration and the desired uniform distribution of p-values under the null for the larger *n*.

Solely to check calibration of the exact placement test while dampening the discrete effects, we created a Monte Carlo version of the exact test (Methods) into which we could additionally add smoothing through adding noise to the sampled clonal exclusivity rates. Performing such smoothing (with *ν* = 10) we observe uniformity of the p-values under the null for larger *n*, with some remnants of the discrete effects visible for lower *n* (Fig 4). The simulated data therefore show that the exact test is calibrated on average.

**Fig 5:**
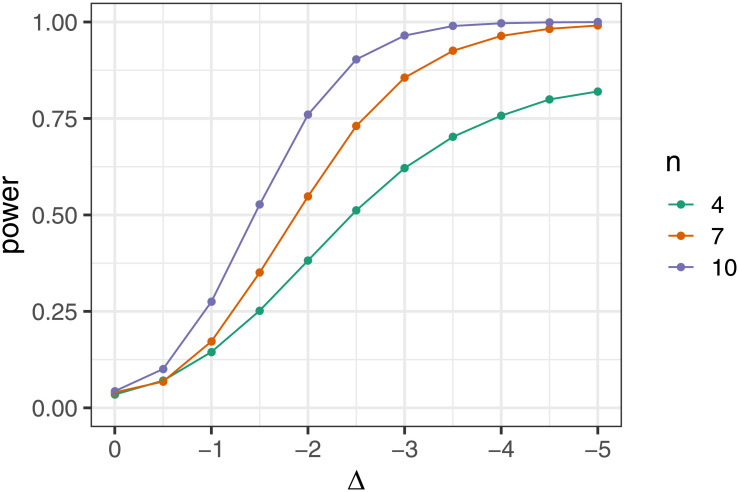
Power of the exact gene pair placement test. The power of the exact placement test as we increase the effect size Δ, which is the change in clonal exclusivity rate on the logit scale, for different sample sizes.

Having checked the calibration of the GeneAccord exact placement test under the null, we next evaluate the power of a single test by simulating data under the alternative for different values of Δ. The rates are again sampled from a beta distribution with parameters 2 and 3. For very small sample sizes, we need a relatively strong effect to have a high power, for example a Δ of -4 to have a power of 75% for gene pairs in only 4 patients. To illustrate this effect size, for the expected exclusivity rate of 40% of the null beta distribution, a shift of Δ = -4 corresponds to an exclusivity rate of 97% for the alternative. The power rapidly increases for larger samples sizes as expected; for example, a Δ of -3 (corresponding to an exclusivity rate of 93% for the alternative compared to 40% for the null) can be detected in 10 patients with a probability of over 96% (Fig 5).

### Comparison to exclusivity testing without trees

Alternative methods to GeneAccord to test for exclusivity do not take the phylogenetic information into account. As a demonstration that utilising evolutionary histories is necessary, we perform naïve exclusivity testing at the clonal level by constructing a contingency table for each gene pair and running standard independence tests: the Fisher’s exact test, the G-test using the chi-squared distribution and the log odds ratio test with the normal approximation. To generate data we uniformly sampled random binary trees each with 10 inner branches and uniformly distributed 20 mutations across those branches to create 10 clonal genotypes per tree. We collated sets of 10 trees, corresponding to gene pairs observed in 10 patient samples, and ran the standard independence tests along with the GeneAccord gene pair placement test. The entire procedure was repeated 400 times.

**Fig 6:**
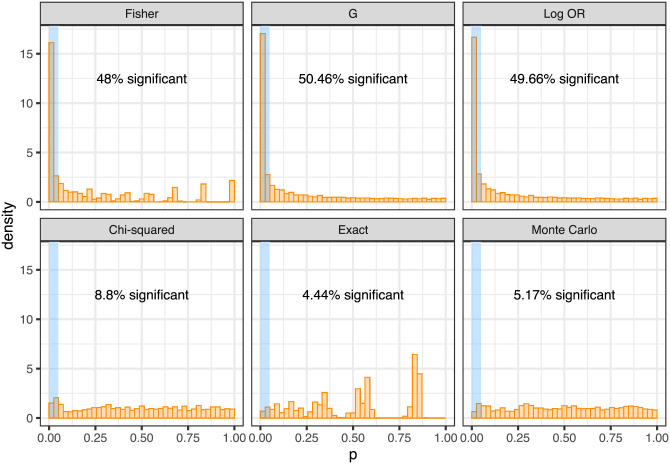
Comparison to standard exclusivity tests. Standard independence tests (top row: Fisher’s exact test, G-test and log odds ratio (OR) test, from left to right) are heavily miscalibrated for data generated from placing mutations on lineage trees under the null. GeneAccord’s gene pair placement test (bottom row: chi-squared approximation, exact test and with Monte Carlo smoothing, from left to right), apart from some enrichment with the chi-squared approximation, shows proper calibration.

As the mutations are interchangeable in their random placement in the trees, we are in the null setting of no clonal enrichment. The standard tests, however, are very heavily confounded and find significant results (at the 5% level) roughly half the time (Fig 6, top row). Not taking the underlying tree structure into account, and treating the clonal genotypes as independent observations, therefore leads to spurious co-occurrence and exclusivity patterns. GeneAccord’s exact placement test remains properly calibrated, as evidenced with the Monte Carlo smoothing, while there is a slight enrichment with the chi-squared approximation (Fig 6, bottom row).

### Gene pair occurrence testing

The GeneAccord gene pair placement test conditions on the set of patients exhibiting a gene pair and their tree topologies. However, gene pairs with a predilection for clonal co-occurrence or exclusivity may occur preferentially in more linear or more branched topologies. We therefore developed the GeneAccord gene pair occurrence test (Methods) to look at whether the set of patients exhibiting mutations in a particular gene pair is indicative of clonal co-occurrence or exclusivity. Testing in which patient set the pair of mutations occurs relies on the chi-squared approximation, but by resampling (with replacement) the trees of the AML cohort and placing genes uniformly among them under the null, we could observe that the approximation is on the conservative side (Fig A in S1 Supplement).

### Combined gene pair clonal co-occurrence and exclusivity testing

Finally we jointly consider the set of patients in which the gene pair occur and the placement of the gene pair within the tree topologies of those patients. For the combined test of the patient set exhibiting mutations and the clonal exclusivity patterns within those patients we could again develop an exact test (Methods). For the simulation based on resampling the AML cohort and placing genes under the null, there are strong discrete effects and conservative p-values for lower significance levels (Fig B in S1 Supplement).

### AML gene pairs

We ran the GeneAccord exact combined test on the cohort of 123 AML patient samples from [12] whose evolutionary histories have been reconstructed with the single-cell phylogeny method SCITE [39]. The results, summarised in Table 1, show 1 gene pair with significant evidence of clonal co-occurrence and 8 with clonal exclusivity.

**Table 1:** GeneAccord combined results for the AML cohort [12]. Ranked list of the gene pairs tested with the GeneAccord exact combined test on the cohort of 123 AML patient samples. For each gene pair, the column *n*_t_ is the total number of patients exhibiting both gene mutations, *n* the number of those patients whose trees are not linear or star shaped and *n*_cx_ the number of times the genes are clonally exclusive within those *n* trees. The columns *n*_l_ and *n*_s_ contain the number of linear and star trees. Δ is the clonal exclusivity score indicating enrichment of clonal co-occurrence (positive) or clonal exclusivity (negative) with ±∞ corresponding to hitting the numerical optimisation bounds. LLR is the log-likelihood ratio statistic, p is the p-value and q the adjusted p-value after Benjamini-Hochberg correction. Only gene pairs with *n* > 3 are considered.

Rank	Gene pair	*n*_t_	*n*	*n*_cx_	*n*_l_	*n*_s_	Δ	LLR	p	q
1	*FLT3*_*NRAS*	15	8	7	3	4	-2.906	26.048	0.00000029	0.0000061
2	*NRAS*_*PTPN11*	9	8	8	1	0	-3.880	24.056	0.0000012	0.000013
3	*KRAS*_*NRAS*	13	9	8	3	1	-2.642	19.289	0.0000090	0.000063
4	*IDH1*_*IDH2*	4	4	4	0	0	-∞	14.577	0.00034	0.0014
5	*KRAS*_*PTPN11*	4	4	4	0	0	-∞	14.577	0.00034	0.0014
6	*FLT3*_*KRAS*	7	4	4	2	1	-2.632	10.704	0.00086	0.0030
7	*FLT3*_*PTPN11*	8	7	5	1	0	-2.227	8.850	0.0021	0.0062
8	*FLT3*_*NPM1*	23	11	0	12	0	∞	8.516	0.0081	0.021
9	*PTPN11*_*WT1*	5	5	3	0	0	-2.069	4.871	0.018	0.043
10	*DNMT3A*_*FLT3*	15	4	0	11	0	∞	5.520	0.036	0.076
11	*NPM1*_*PTPN11*	11	8	0	3	0	∞	4.041	0.087	0.17
12	*DNMT3A*_*NPM1*	18	5	1	13	0	1.250	2.053	0.16	0.28
13	*KRAS*_*NPM1*	7	5	0	2	0	∞	2.569	0.23	0.37
14	*IDH2*_*NRAS*	10	6	3	4	0	-0.812	1.171	0.30	0.45
15	*IDH2*_*NPM1*	12	7	1	5	0	0.797	0.701	0.36	0.49
16	*IDH2*_*PTPN11*	4	4	0	0	0	∞	1.468	0.37	0.49
17	*DNMT3A*_*IDH2*	11	4	1	7	0	0.699	0.520	0.55	0.68
18	*NPM1*_*NRAS*	18	11	2	7	0	0.468	0.413	0.63	0.73
19	*FLT3*_*IDH1*	5	4	1	1	0	-0.230	0.040	0.80	0.81
20	*FLT3*_*IDH2*	8	5	1	3	0	0.335	0.104	0.81	0.81
21	*IDH1*_*NPM1*	8	5	1	3	0	0.335	0.104	0.81	0.81

One clonally exclusive gene pair is between the two *IDH* genes, while 6 others involve the genes *FLT3*, *NRAS*, *KRAS* and *PTPN11*, which affect the receptor tyrosine kinase (RTK)/Ras GTPase (RAS)/MAP Kinase (MAPK) signalling pathways. Clonal exclusivity would align with functional redundancy making the mutations interchangeable, though having several mutations in parallel lineages is evolutionarily more complex than sharing a single mutation in an ancestral clone. The significant clonal exclusivity of these gene pairs may then point to stronger effects like cooperation across the clones or synthetic lethality where having co-occurring mutations in the same lineage leads to strong decrease in viability of the tumour clone [40, 41].

We observe significant co-occurrence of *NPM1*, the most frequent mutation in the cohort, with *FLT3*. There is a strong interplay between *NPM1*, *FLT3* and age in terms of survival prognosis [42], and proven biological cooperation in AML between the genes [43–45].

The combined test accounts for both the placement of mutations within the trees of patients with both mutations in a gene pair and the occurrence of the mutations across the patient cohort. When considering just the set of patients exhibiting each gene pair with the gene pair occurrence test (Table A in S1 Supplement) we can test whether the gene pair is over enriched in more linear or more branching trees compared to chance. Since the majority of the AML cohort have linear trees, co-occurrence is less surprising under the null, but we do observe some clonally exclusive pairs enriched in the more branched trees, particularly *FLT3* and *NRAS* which appear in 4 out of the 5 star trees in the cohort.

**Fig 7:**
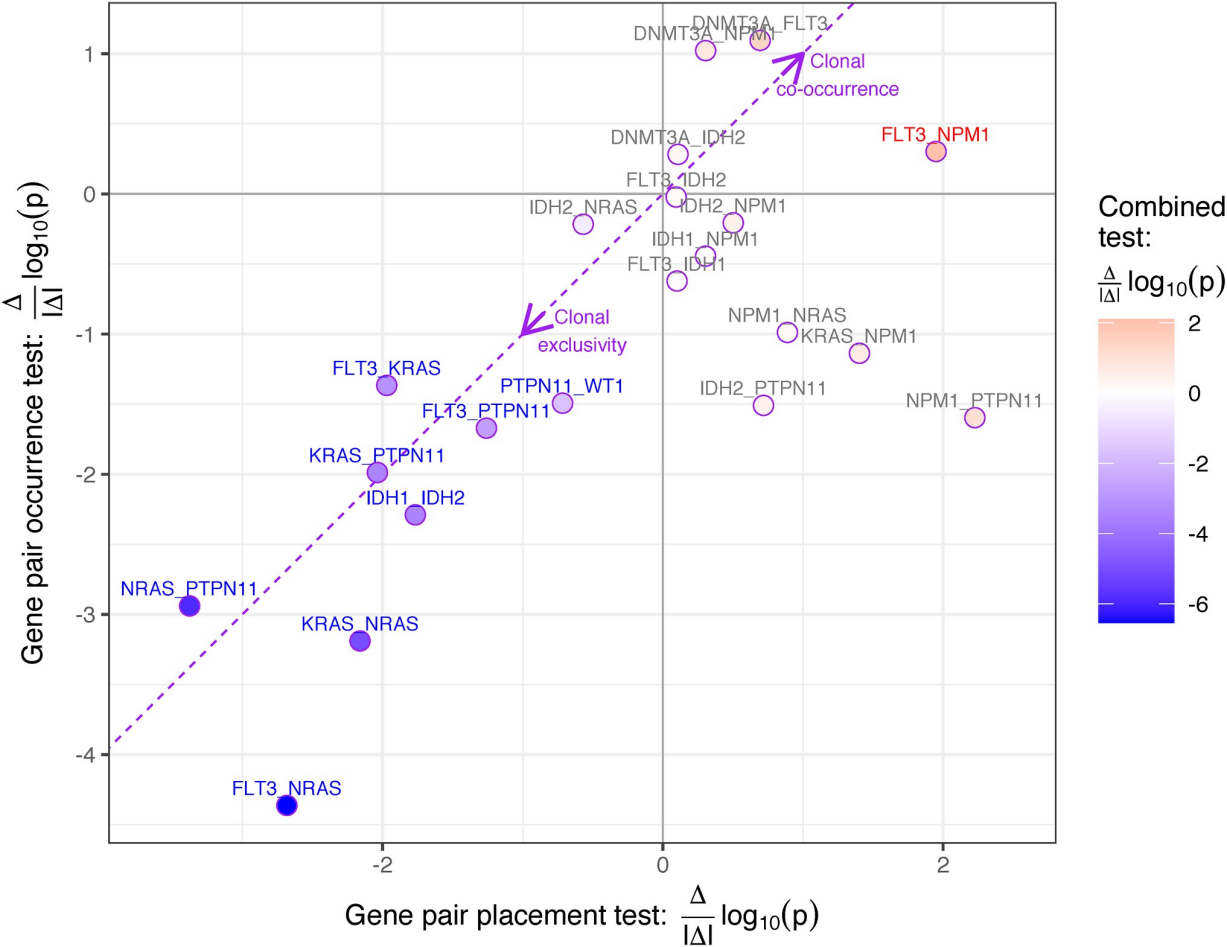
Significance of clonal co-occurrence or exclusivity of the three GeneAccord tests for the AML cohort. For the AML cohort, we plot the significance of clonal co-occurrence or exclusivity by computing the log_10_ p-value and including the sign of the effect $\frac{\Delta }{\left|\Delta \right|}$ (positive indicating clonal co-occurrence, negative indicating clonal exclusivity) for the three geneAccord tests: The x-axis depicts the placement test, the y-axis the occurrence test and the colouring the combined test (red indicating clonal co-occurrence, blue indicating clonal exclusivity). The labels of the significant gene pairs of the combined test, after Benjamini-Hochberg correction (Table 1), are also coloured.

Considering just the placement of mutations within the patient trees exhibiting both mutations with the gene pair placement test (Table B in S1 Supplement), we find 6 of the 8 clonally exclusive pairs and the clonally co-occurring pair from the combined test (Table 1). Additionally we also find significant clonal co-occurrence of the pair *NPM1* and *PTPN11* when looking only at the placement of the mutations within patient trees, but this is not corroborated by the gene pair occurrence test (Table A in S1 Supplement) and hence not significant for the combined test. In general, the combined test merges the signals from the gene pair placement and occurrence tests and increases the significance of the clonally exclusive pairs (Fig 7).

For comparison, if we run standard mutual exclusivity testing at the clone level and ignore the phylogenies, then despite the enrichment of p-values under the null (Fig 6), only 5 gene pairs would be considered significant (Table C in S1 Supplement). These include pairs found by GeneAccord particularly *FLT3* being clonally exclusive with *NRAS* and clonally co-occurring with *NPM1*. Notably, the naïve test assigns significance to exclusivity between *FLT3* and *IDH2* while this pair fits well with random assignment across the patient cohort under the null as indicated by the GeneAccord analysis, and if anything with a tendency to co-occur clonally (Table 1). The pair of *IDH1* and *NPM1* picked up by the naïve testing is also unremarkable when considering the patient tree topologies with GeneAccord.

## Discussion

We have introduced GeneAccord as a novel statistical framework to systematically analyse the subclonal mutation patterns in cohorts of tumour patients. We first considered the placement of the mutations within the evolutionary histories of the patients exhibiting both mutations. By introducing a null model of random mutation placement, we created a new exact test to assess how unusual the subclonal patterns are across the cohort. We evaluated the calibration and power of the test. For larger numbers of patient samples exhibiting the same pair of genes, the exact test becomes more computationally intensive, while utilising the asymptotic chi-squared distribution becomes better calibrated and is computationally cheap. Such testing conditions on the observed tumour phylogenies, so that linear and star shaped trees are not informative for the placement test. However, linear and star topologies may be favoured with clonally co-occurring or exclusive gene pairs. To account for these signals we developed a gene pair occurrence test for which patients exhibit both mutations. We then created a combined test of both the occurrence of mutations across the cohort and the placement within the patient trees carrying both mutations. This allows us to utilise all data to better uncover clonal co-occurrence and exclusivity.

Though the combined test additionally utilises the signals of where a gene pair occurs across the set of tumour phylogenies, the testing framework is conditioned on this set of trees. If all the trees are linear (or all star-shaped) then all gene pairs are clonally co-occurring (or exclusive) and hence interchangeable, which defines our null model. As such, GeneAccord relies on there being a mixture of tree topologies in the cohort to be able to detect significant pairs. We focussed on clonal co-occurrence and exclusivity at the gene level, but the method applies to any marker that can be assigned to the clones in the architecture of each tumour, for example to pathways by mapping from mutations to pathways [46] or from differentially expressed genes to pathways.

We detect pairs of genes or pathways that have an elevated or depressed rate of mutating in different clonal lineages of the same tumour. There are several possible biological explanations for such mutational patterns. For clonally exclusive gene pairs occurring in separate co-existing clones, the two clones could have complementing phenotypes and cooperate or mutually benefit each other, for instance, by sharing diffusible factors. Another possibility is that the two genes of a clonally exclusive pair are synthetically lethal and that both genes being mutated in the same cell would lead to a disadvantageous, and maybe even lethal, phenotype. The two clones may also be the result of parallel convergent evolution where both clones exhibit the same phenotype by different mutations. The specific subclonal mutation pattern may also depend on the evolutionary subtype [9]. In order to gain a better understanding of possible reasons for the clonally exclusive pattern, it is important to examine the biological functions of these genes and pathways in more detail. A definite proof of such interactions then requires future experimental studies in vitro or in vivo. Here, we have developed and applied a new computational approach to identify such gene (or pathway) pairs that are unlikely to be generated by random chance alone. As such, the GeneAccord method will be useful in finding and prioritising candidate cooperative tumour clones in cancer patient cohorts.

While many cancer-related mutually exclusive gene pairs have been previously identified across cohorts at a bulk or consensus level, finding such pairs within a single tumour and its clonal architecture has received less attention. Especially with the high resolution of multi-region bulk and single-cell sequencing we can now better reconstruct the evolution histories of tumours for downstream analyses as performed here. As the panels used for high-throughput single-cell sequencing increase in size and coverage we can expect more detailed tumour phylogenies and better clonal resolution to provide more power to detect clonal exclusivity patterns. As a future extension we could integrate our testing with evolutionary modelling to extract additional signals of subclonal cooperation. Currently we focused on testing genes pairwise, and another possible extension would be to consider larger sets of genes and detect more generalised (higher order) clonal exclusivity patterns.

Looking ahead, combined single-cell sequencing of the exome and transcriptome [47] will allow the assignment of differentially expressed genes to specific clones. Alternatively, matching multi-omic profiling of the different cells from the same sample, for example, through matching expression profiles and copy number states [48] on evolutionary trees [49], would provide a transcriptomic profile for each clone. This would enable a GeneAccord analysis on the transcriptomic level. Therefore, with single-cell data, one could perform a combined analysis using various omics layers including differentially expressed genes, as well as copy number and epigenetic changes. This would allow for a more holistic portrait of the subclonal alteration profiles and potentially reveal more synergies between clones that could inform the design of future treatments.

## Methods

### GeneAccord overview

The input data to the GeneAccord algorithm are the mutated gene-to-clone assignments from a cohort of cancer patients. These are obtained by running phylogenetic tree inference methods, for example on single-cell sequencing data. We utilised the trees inferred by SCITE [39] for the cohort of 123 AML patient samples [12].

In general, there is uncertainty in the tree structure learned from sequencing data. Therefore, our algorithm was designed to allow as input multiple gene-to-clone assignments per patient, for example by sampling from the posterior distribution of trees. The tree inference designates point mutations to individual clones, while mutations can then be mapped to genes or pathways using existing pathway databases. Our statistical framework can be applied on the gene level, or on the pathway level to detect clonally exclusive pairs of pathways. We focus on the gene case here.

### Likelihood ratio gene pair placement test

To test if gene pairs have a different rate of clonal exclusivity compared to typical genes, we first compute the background rate of clonal exclusivity for each patient *i*

$$\begin{array}{c} r_{i}=\frac{\#\;\text{gene}\;\text{pairs}\;\text{on}\;\text{different}\;\text{branches}}{\#\;\text{gene}\;\text{pairs}} \end{array}$$ (1)

When we have a sample of trees for the patient, we average this rate across the trees. Then for each gene pair (*j*, *k*), we look at the clonal exclusivity of gene *j* and gene *k* among the patient samples that possess both somewhere in their trees. For each patient *i*, we compute

$$\begin{array}{c} O_{i}^{\left(j,k\right)}=\frac{\#\;\text{trees}\;\text{with}\;\text{gene}\;j\;\text{and}\;\text{gene}\;k\;\text{clonally}\;\text{exclusive}}{\#\;\text{trees}} \end{array}$$ (2)

When we have a single tree for patient *i*, this quantity will be either 0 or 1. Under the null model that genes are placed randomly on the trees for each patient, the likelihood that a gene pair will be exclusive is *r*_*i*_ while the likelihood of co-occurrence is (1 − *r*_*i*_). The log-likelihood of the observed exclusivity patterns of the gene pair is then

$$\begin{array}{c} l_{0}^{\left(j,k\right)}=\sum_{i=1}^{n}[O_{i}^{\left(j,k\right)}\;\text{log}\left(r_{i}\right)+(1-O_{i}^{\left(j,k\right)})\;\text{log}\left(1-r_{i}\right)] \end{array}$$ (3)

where the sum is only over patients with both genes present, with *n* denoting the number of patient samples with both genes. For the alternative model, we allow the clonal exclusivity rates to differ for the gene pair. To ensure that the shifted rates are between 0 and 1, we perform the shift in the logit space and define the shifted rate $r_{i}^{\prime }=r_{i}^{\prime }\left(\Delta_{p}\right)$ by

$$\begin{array}{c} \text{logit}\left(r_{i}^{\prime }\right)=\text{logit}\left(r_{i}\right)-\Delta_{p}\;,\;\text{logit}\left(x\right)=\text{log}(\frac{x}{1-x}) \end{array}$$ (4)

where the sign of the shift Δ_p_ is chosen so that positive values indicate co-occurrence and negative values indicate clonal exclusivity. Then we maximise the log-likelihood of the alternative over Δ_p_

$$\begin{array}{c} l_{1}^{\left(j,k\right)}=\underset{\Delta_{p}}{\text{max}}\sum_{i=1}^{n}[O_{i}^{\left(j,k\right)}\;\text{log}\left(r_{i}^{\prime }\right)+(1-O_{i}^{\left(j,k\right)})\;\text{log}\left(1-r_{i}^{\prime }\right)] \end{array}$$ (5)

where the dependence on Δ_p_ is through the *r*′ and the maximisation is performed numerically (with the optimize function in R). For computational reasons, we restrict Δ_p_ to a range of ±10 since after the logit transformations the *r*′ will essentially be 0 or 1 and further increasing or decreasing Δ to ±∞ will hardly affect the maximal log-likelihood numerically. As a test statistic we employ the log-likelihood ratio (LLR)

$$\begin{array}{c} {\text{LLR}}_{p}=2(l_{1}^{\left(j,k\right)}-l_{0}^{\left(j,k\right)}) \end{array}$$ (6)

The factor of 2 is included so that for larger *n* the LLR statistic will follow a $\chi_{1}^{2}$ distribution. For linear trees all gene pairs are in the same lineage and with none being clonally exclusive *r*_*i*_ = 0. The logit transform maps to -∞ which is unaffected by the shift of Δ_p_ and the transformed rate $r_{i}^{\prime }$ will also be 0. Similarly, for star trees with every gene in its own lineage and *r*_*i*_ = 1, the shifted rates are unchanged. These topologies therefore do not contribute to the LLR statistic for the gene pair placement test, and patients with such uninformative trees are removed from the test.

### Exact placement test

To be able to use GeneAccord for less common gene pairs we devise an exact test for smaller *n*. When the gene pair is observed in *n* patient samples, there are 2^*n*^ different possible binary clonal exclusivity patterns. If we store the binary pattern in a vector ***b***, the probability of it arising under the null is

$$\begin{array}{c} P\left(b\right)=\prod_{i=1}^{n}[r_{i}b_{i}+\left(1-r_{i}\right)\left(1-b_{i}\right)] \end{array}$$ (7)

For each binary vector, we compute its LLR statistic. The p-value is the sum of the probabilities of the binary vectors with a LLR statistic larger than the observed statistic. In the p-value we also include half the probabilities of binary vectors with identical LLR statistics. This is necessary to obtain calibration of the p-values, which we demonstrate with Monte Carlo smoothing.

### Monte Carlo smoothing

To better check the calibration of the exact test we wish to introduce some smoothing into the p-value distribution. Rather than enumerating all binary vectors, we could simply sample them proportionally to their probabilities and obtain a Monte Carlo estimate of the p-value. In the limit of an infinite sample size this reduces to the value from the exact test, with the same discrete effects. To smooth these effects we add some noise to the values of *r* for each Monte Carlo sample by sampling them from a beta distribution with parameters *νr* and *ν*(1 − *r*). The parameter *ν* corresponds to the amount of overdispersion or noise in the sampled rates with the limit *ν* → ∞ being noiseless. From the Monte Carlo samples, the p-value is the proportion of samples with a larger LLR statistic (the probability of being equal is 0). As we take the limit *ν* → ∞ the median of a beta distribution approaches the mean, so that half the Monte Carlo samples which would have the same LLR in the exact test will be more extreme, and half less extreme. In the exact test we therefore count binary vectors with the same discrete LLR statistic with weight half.

### Computational cost

After the data pre-processing, computing the LLR statistic involves optimising the log-likelihood numerically while each likelihood computation is linear in *n*, the number of patient samples. For the chi-squared approximation we perform a single optimisation so for each gene pair considered the cost is *O*(*n*). The exact test involves optimising 2^*n*^ log-likelihoods leading to a cost of *O*(*n*2^*n*^). The Monte Carlo smoothed version optimises for each Monte Carlo sample, so with *M* samples the cost is *O*(*nM*).

### Alpha budgeting

For gene pairs mutated in only a handful of patient samples, depending on the background clonal exclusivity rates for those patients, it may never be possible to get a significant p-value below 5% regardless of the observed clonal exclusivity patterns in the data. For each gene pair we therefore compute, from the set of patient samples possessing both mutations, the minimum possible p-value. If the minimum is greater than 5%, the gene pair is removed before the testing with GeneAccord so as not to affect multiple testing corrections.

### Gene pair occurrence test

In the GeneAccord gene pair placement test, we condition on the set of patient samples that exhibit the gene pair under consideration. In a linear (star) tree all gene pairs are clonally co-occurring (exclusive) under the null and cannot change under the alternative so that these topologies are uninformative for the test. To enrich GeneAccord, we can additionally consider whether these topologies are over or under-enriched and hence whether the set of tumour samples themselves is indicative of clonal co-occurrence or exclusivity. For this we develop the following gene pair occurrence test.

Under the null model that all gene pairs are alike and interchangeable, the probability a gene pair occurs in a specific patient is proportional to the number of gene pairs it possesses. Let *w*_*i*_ be the number of gene pairs of patient *i*. Then the probability a set *S* of patients exhibits the gene pair is

$$\begin{array}{c} P_{0}^{S}=\frac{1}{Z}\prod_{i\in S}w_{i}\;,\;Z=\sum_{S^{\prime }}^{|S^{\prime }\left|=|S\right|}\prod_{i\in S^{\prime }}w_{i} \end{array}$$ (8)

where we condition on the size of the set and normalise over all possible patient sets of that size (this is the non-central hypergeometric distribution in the Fisher rather than Wallenius sense; [50]). Although for a dataset with *N* patients, evaluating the normalising constant *Z* involves $\begin{array}{c} \left(\begin{array}{c} N\\ \left|S\right| \end{array}\right) \end{array}$ possible sets, with dynamic programming we compute *Z* in *O*(*N*|*S*|) time.

As an alternative, we might expect gene pairs with a propensity for clonal co-occurrence (exclusivity) to prefer topologies with fewer (more) branches so we allow the weight of a patient to depend on its clonal exclusivity rate

$$\begin{array}{c} w_{i}^{\prime }=2w_{i}[\rho \left(1-r_{i}\right)+\left(1-\rho \right)r_{i}]\;,\;\text{logit}\left(\rho \right)=\Delta_{o} \end{array}$$ (9)

This formula gives a relative weight of *ρ* to locations in the tree of clonal co-occurrence and a relative weight of (1 − *ρ*) to clonally exclusive placements of the gene pair. With the logit transformation, Δ_o_ measures the degree of clonal co-occurrence (positive) or exclusivity (negative) analogously to the GeneAccord placement test. For the alternative we numerically maximise the probability of selecting the observed patients

$$\begin{array}{c} P_{1}^{S}=\underset{\Delta_{o}}{\text{max}}\frac{1}{Z^{\prime }}\prod_{i\in S}w_{i}^{\prime }\;,\;Z^{\prime }=\sum_{S^{\prime }}^{|S^{\prime }\left|=|S\right|}\prod_{i\in S^{\prime }}w_{i}^{\prime } \end{array}$$ (10)

to obtain the LLR test statistic

$$\begin{array}{c} {\text{LLR}}_{o}=2(\text{log}\;P_{1}^{S}-\text{log}\;P_{0}^{S}) \end{array}$$ (11)

To enumerate and maximise all $\begin{array}{c} \left(\begin{array}{c} N\\ \left|S\right| \end{array}\right) \end{array}$ possible patient sets to obtain an exact test is computationally prohibitive, so here we rely on the $\chi_{1}^{2}$ approximation for the distribution of the LLR statistic for larger |*S*|.

### Combined test

Since the GeneAccord gene pair placement and occurrence tests are independent under the null, the p-values from each can be combined with Fisher’s method to obtain a joint p-value. To better account for consistent signals, we instead compute the joint probability of the gene pair (*j*, *k*) occurring in the selected patient set *S* and their clonal exclusivity patterns. The joint log-likelihood can be written as

$$\begin{array}{cc} & \sum_{i\in S}[\text{log}\;(w_{i}^{\prime })+O_{i}^{\left(j,k\right)}\text{log}\;(\frac{\left(1-\rho \right)r_{i}}{\rho \left(1-r_{i}\right)+\left(1-\rho \right)r_{i}})\\ & \;+(1-O_{i}^{\left(j,k\right)})\;\text{log}\;(\frac{\rho (1-r_{i})}{\rho \left(1-r_{i}\right)+\left(1-\rho \right)r_{i}})]-\text{log}\;\left(Z^{\prime }\right) \end{array}$$ (12)

and if we equate the two shift parameters Δ = Δ_p_ = Δ_*o*_ we have the relationship

$$\begin{array}{c} \frac{\left(1-\rho \right)r_{i}}{\rho \left(1-r_{i}\right)+\left(1-\rho \right)r_{i}}=r_{i}^{\prime }\;,\;\frac{\rho (1-r_{i})}{\rho \left(1-r_{i}\right)+\left(1-\rho \right)r_{i}}=\left(1-r_{i}^{\prime }\right) \end{array}$$ (13)

so that the joint log-likelihood simply reduces to combining the log-likelihoods of the placement and occurrence tests.

$$\begin{array}{c} {\text{LLR}}_{c}={\text{LLR}}_{p}+{\text{LLR}}_{o}\;,\;\Delta =\Delta_{p}=\Delta_{o} \end{array}$$ (14)

When we maximise and compute the difference in log-likelihoods to obtain the LLR statistic, the weights of the selected patients in *S* actually cancel. For a given cohort, the LLR is then determined by the number of trees where the gene pair is clonally exclusive (or co-occurring) including the linear and star trees. Since this number can range from 0 to the total number of patients exhibiting the gene pair, we again enumerate all possibilities and compute their probability under the null to obtain an exact p-value.

### AML cohort processing

In the AML cohort [12], we start with the trees inferred with SCITE [39] and filtered out any clones with a frequency below 1%. Since most patient samples only exhibit a few mutations amongst the panel of 30 genes selected, the majority of trees are linear. Of the 123 patient samples, 3 have a single mutation and no gene pairs, leaving 120 samples. Among these there are 80 linear trees, 5 star trees and 35 which are informative for the GeneAccord gene pair placement test. We then consider gene pairs which were both present in at least 4 patient trees among those 35, leading to a set of 21 gene pairs. These 21 gene pairs are analysed over the full cohort of 120 samples with the combined GeneAccord test (Table 1).

## Supporting information

S1 Supplement Supplementary material. Calibration plots of the GeneAccord gene pair occurrence test and the GeneAccord combined test of clonal co-occurrence or exclusivity. Tables of the results of the GeneAccord gene pair occurrence test, the GeneAccord gene pair placement test and naïve exclusivity testing on the AML cohort. **Fig A. Calibration of the gene pair occurrence test**. For gene pairs simulated under the null to occur in *n* patients the test for which patients exhibit the mutations has some degree of miscalibration with the chi-squared approximation, but is conservative for significant p-values. The simulation is based on resampling the AML trees. **Fig B. Calibration of the combined GeneAccord test**. For the simulation based on resampling the AML trees and placing genes uniformly across them (as in Fig A) we consider gene pairs occurring in *n* patients. The exact test combines the signals from the gene pair occurrence amongst patients with the placement of the mutations within those patients. The test has strong discrete effects, but conservative p-values at lower significance levels. **Table A. GeneAccord gene pair occurrence test results for the AML cohort**. Ranked list of the gene pairs tested with the GeneAccord gene pair occurrence chi-squared test on the cohort of 123 AML patient samples. For each gene pair, the column *n*_t_ is the total number of patients exhibiting both gene mutations. The column *n* contains the number of those patients whose trees are not linear or star shaped while *n*_l_ and *n*_s_ contain the number of linear and star trees. Δ_o_ is the clonal exclusivity score indicating enrichment of clonal co-occurrence (positive) or clonal exclusivity (negative) with ±∞ corresponding to hitting the numerical optimisation bounds. LLR is the log-likelihood ratio statistic, p is the p-value and q the adjusted p-value after Benjamini-Hochberg correction. Only gene pairs with *n* > 3 are considered. **Table B. GeneAccord gene pair placement test results for the AML cohort**. Ranked list of the gene pairs tested with the GeneAccord exact placement test on the cohort of 123 AML patient samples. For each gene pair, the column *n* is the number of patients exhibiting both gene mutations, *n*_cx_ the number of times the genes are clonally exclusive. Linear and star trees which are not informative for the test are excluded. Δ_p_ is the clonal exclusivity score indicating enrichment of clonal co-occurrence (positive) or clonal exclusivity (negative) with ±∞ corresponding to hitting the numerical optimisation bounds. LLR is the log-likelihood ratio statistic, p is the p-value and q the adjusted p-value after Benjamini-Hochberg correction. Only gene pairs with *n* > 3 are included. **Table C. Naïve exclusivity testing on the clones the AML cohort**. Ranked list of the gene pairs tested with standard exclusivity testing on the 492 clones in the cohort of 123 AML patient samples. For testing we compute the log odds ratio and use the normal approximation. For each gene pair, the column z is the z-score indicating enrichment of clonal co-occurrence (positive) or clonal exclusivity (negative), p is the p-value and q the adjusted p-value after Benjamini-Hochberg correction. Only gene pairs tested with GeneAccord are considered.
(PDF)
