# Supplementary material for: Statistical tests for intra-tumour clonal co-occurrence and exclusivity
Source: PLoS Comput Biol. 2021 Dec 15;17(12):e1009036. doi: 10.1371/journal.pcbi.1009036 (PMC8716063; doi:10.1371/journal.pcbi.1009036)
Supplement: S1 Supplement — Calibration plots of the GeneAccord gene pair occurrence test and the GeneAccord combined test of clonal co-occurrence or exclusivity. Tables of the results of the GeneAccord gene pair occurrence test, the GeneAccord gene pair placement test and naïve exclusivity testing on the AML cohort. Fig A. Calibration of the gene pair occurrence test. For gene pairs simulated under the null to occur in n patients the test for which patients exhibit the mutations has some degree of miscalibration with the chi-squared approximation, but is conservative for significant p-values. The simulation is based on resampling the AML trees. Fig B. Calibration of the combined GeneAccord test. For the simulation based on resampling the AML trees and placing genes uniformly across them (as in Fig A) we consider gene pairs occurring in n patients. The exact test combines the signals from the gene pair occurrence amongst patients with the placement of the mutations within those patients. The test has strong discrete effects, but conservative p-values at lower significance levels. Table A. GeneAccord gene pair occurrence test results for the AML cohort. Ranked list of the gene pairs tested with the GeneAccord gene pair occurrence chi-squared test on the cohort of 123 AML patient samples. For each gene pair, the column nt is the total number of patients exhibiting both gene mutations. The column n contains the number of those patients whose trees are not linear or star shaped while nl and ns contain the number of linear and star trees. Δo is the clonal exclusivity score indicating enrichment of clonal co-occurrence (positive) or clonal exclusivity (negative) with ±∞ corresponding to hitting the numerical optimisation bounds. LLR is the log-likelihood ratio statistic, p is the p-value and q the adjusted p-value after Benjamini-Hochberg correction. Only gene pairs with n > 3 are considered. Table B. GeneAccord gene pair placement test results for the AML cohort. Ranked list of the gene pairs teste [file pcbi.1009036.s001.pdf]

Supplementary Material for
Statistical tests for intra-tumour clonal co-occurrence and exclusivity

Supplementary Figures and Tables

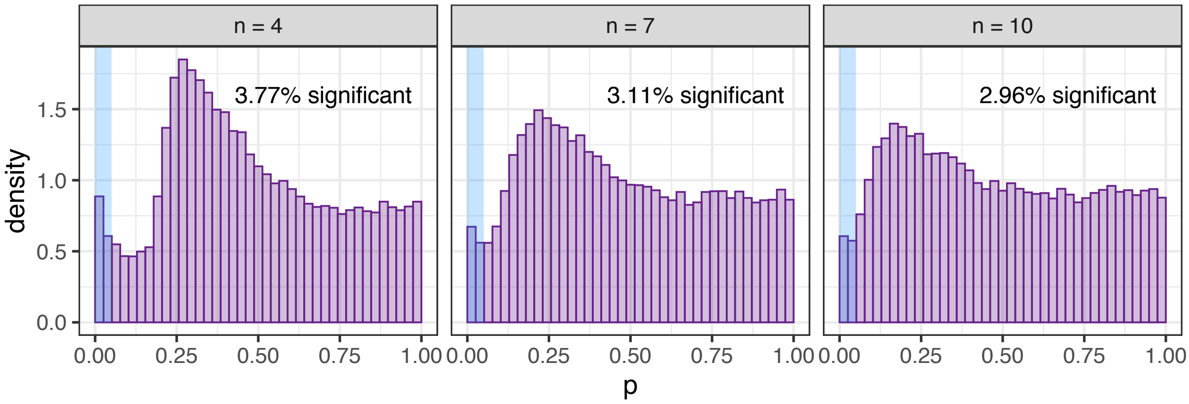

Fig A: **Calibration of the gene pair occurrence test.** For gene pairs simulated under the null to occur in n patients the test for which patients exhibit the mutations has some degree of miscalibration with the chi-squared approximation, but is conservative for significant p-values. The simulation is based on resampling the AML trees.

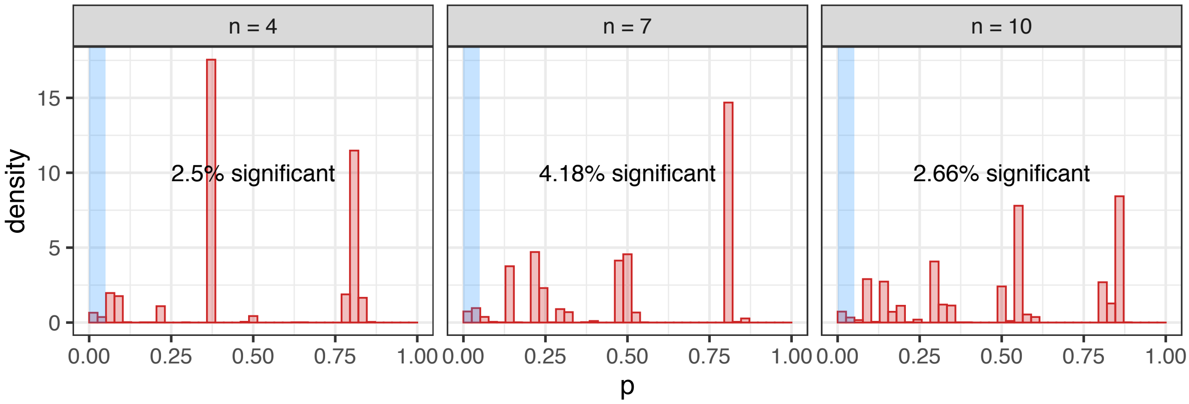

Fig B: **Calibration of the combined GeneAccord test.** For the simulation based on resampling the AML trees and placing genes uniformly across them (as in Fig A) we consider gene pairs occurring in n patients. The exact test combines the signals from the gene pair occurrence amongst patients with the placement of the mutations within those patients. The test has strong discrete effects, but conservative p-values at lower significance levels.

Table A: **GeneAccord gene pair occurrence test results for the AML cohort.** Ranked list of the gene pairs tested with the GeneAccord gene pair occurrence chi-squared test on the cohort of 123 AML patient samples. For each gene pair, the column n_t is the total number of patients exhibiting both gene mutations. The column n contains the number of those patients whose trees are not linear or star shaped while n_l and n_s contain the number of linear and star trees. Δ_o is the clonal exclusivity score indicating enrichment of clonal co-occurrence (positive) or clonal exclusivity (negative) with $\pm\infty$ corresponding to hitting the numerical optimisation bounds. LLR is the log-likelihood ratio statistic, p is the p-value and q the adjusted p-value after Benjamini-Hochberg correction. Only gene pairs with $n > 3$ are considered.

Rank	Gene pair	n_t	n	n_l	n_s	Δ_o	LLR	p	q
1	*FLT3_NRAS*	15	8	3	4	-2.986	16.715	0.000043	0.00091
2	*KRAS_NRAS*	13	9	3	1	-2.729	11.636	0.00065	0.0068
3	*NRAS_PTPN11*	9	8	1	0	-3.486	10.569	0.0011	0.0080
4	*IDH1_IDH2*	4	4	0	0	$-\infty$	7.832	0.0051	0.027
5	*KRAS_PTPN11*	4	4	0	0	$-\infty$	6.584	0.010	0.043
6	*FLT3_PTPN11*	8	7	1	0	-2.929	5.300	0.021	0.075
7	*NPM1_PTPN11*	11	8	3	0	-2.236	5.001	0.025	0.075
8	*IDH2_PTPN11*	4	4	0	0	$-\infty$	4.654	0.031	0.075
9	*PTPN11_WT1*	5	5	0	0	$-\infty$	4.590	0.032	0.075
10	*FLT3_KRAS*	7	4	2	1	-2.224	4.093	0.043	0.090
11	*KRAS_NPM1*	7	5	2	0	-2.157	3.216	0.073	0.14
12	*DNMT3A_FLT3*	15	4	11	0	∞	3.056	0.080	0.14
13	*DNMT3A_NPM1*	18	5	13	0	∞	2.787	0.095	0.15
14	*NPM1_NRAS*	18	11	7	0	-1.429	2.663	0.10	0.15
15	*FLT3_IDH1*	5	4	1	0	-2.151	1.387	0.24	0.33
16	*IDH1_NPM1*	8	5	3	0	-1.293	0.840	0.36	0.47
17	*FLT3_NPM1*	23	11	12	0	1.512	0.458	0.50	0.61
18	*DNMT3A_IDH2*	11	4	7	0	1.233	0.409	0.52	0.61
19	*IDH2_NRAS*	10	6	4	0	-0.778	0.264	0.61	0.65
20	*IDH2_NPM1*	12	7	5	0	-0.722	0.244	0.62	0.65
21	*FLT3_IDH2*	8	5	3	0	-0.154	0.004	0.95	0.95

Table B: **GeneAccord gene pair placement test results for the AML cohort.** Ranked list of the gene pairs tested with the GeneAccord exact placement test on the cohort of 123 AML patient samples. For each gene pair, the column n is the number of patients exhibiting both gene mutations, n_{cx} the number of times the genes are clonally exclusive. Linear and star trees which are not informative for the test are excluded. $\Delta\mathrm{p}$ is the clonal exclusivity score indicating enrichment of clonal co-occurrence (positive) or clonal exclusivity (negative) with $\pm\infty$ corresponding to hitting the numerical optimisation bounds. LLR is the log-likelihood ratio statistic, p is the p-value and q the adjusted p-value after Benjamini-Hochberg correction. Only gene pairs with $n > 3$ are included.

Rank	Gene pair	n	n_{cx}	$\Delta\mathrm{p}$	LLR	p	q
1	*NRAS_PTPN11*	8	8	$-\infty$	14.164	0.00042	0.0088
2	*FLT3_NRAS*	8	7	-2.704	9.378	0.0021	0.022
3	*NPM1_PTPN11*	8	0	∞	9.040	0.0059	0.034
4	*KRAS_NRAS*	9	8	-2.434	7.700	0.0069	0.034
5	*KRAS_PTPN11*	4	4	$-\infty$	7.994	0.0092	0.034
6	*FLT3_KRAS*	4	4	$-\infty$	7.686	0.011	0.034
7	*FLT3_NPM1*	11	0	∞	8.131	0.011	0.034
8	*IDH1_IDH2*	4	4	$-\infty$	6.746	0.017	0.045
9	*KRAS_NPM1*	5	0	∞	5.862	0.040	0.092
10	*FLT3_PTPN11*	7	5	-1.756	4.164	0.055	0.115
11	*NPM1_NRAS*	11	2	1.321	2.917	0.129	0.246
12	*IDH2_PTPN11*	4	0	∞	3.122	0.191	0.303
13	*PTPN11_WT1*	5	3	-1.469	2.028	0.193	0.303
14	*DNMT3A_FLT3*	4	0	∞	2.464	0.202	0.303
15	*IDH2_NRAS*	6	3	-0.828	0.908	0.270	0.379
16	*IDH2_NPM1*	7	1	1.098	1.247	0.314	0.413
17	*DNMT3A_NPM1*	5	1	0.618	0.326	0.495	0.578
18	*IDH1_NPM1*	5	1	0.847	0.635	0.496	0.578
19	*DNMT3A_IDH2*	4	1	0.521	0.179	0.780	0.804
20	*FLT3_IDH1*	4	1	0.297	0.066	0.791	0.804
21	*FLT3_IDH2*	5	1	0.407	0.137	0.804	0.804

Table C: **Naïve exclusivity testing on the clones the AML cohort.** Ranked list of the gene pairs tested with standard exclusivity testing on the 492 clones in the cohort of 123 AML patient samples. For testing we compute the log odds ratio and use the normal approximation. For each gene pair, the column z is the z-score indicating enrichment of clonal co-occurrence (positive) or clonal exclusivity (negative), p is the p-value and q the adjusted p-value after Benjamini-Hochberg correction. Only gene pairs tested with GeneAccord are considered.

Rank	Gene pair	z	p	q
1	*NPM1_PTPN11*	3.820	0.000013	0.0028
2	*FLT3_NRAS*	-3.424	0.00062	0.0065
3	*FLT3_NPM1*	2.645	0.0082	0.043
4	*FLT3_IDH2*	-2.591	0.010	0.043
5	*IDH1_NPM1*	2.567	0.010	0.043
6	*NRAS_PTPN11*	-2.173	0.030	0.10
7	*IDH1_IDH2*	-2.135	0.033	0.10
8	*IDH2_NRAS*	-1.849	0.064	0.16
9	*FLT3_PTPN11*	-1.835	0.066	0.16
10	*FLT3_KRAS*	-1.681	0.093	0.19
11	*FLT3_IDH1*	-1.595	0.11	0.21
12	*DNMT3A_FLT3*	1.558	0.13	0.21
13	*KRAS_PTPN11*	-1.211	0.23	0.35
14	*KRAS_NPM1*	1.184	0.24	0.35
15	*IDH2_NPM1*	0.959	0.34	0.47
16	*DNMT3A_NPM1*	0.728	0.47	0.61
17	*DNMT3A_IDH2*	-0.566	0.57	0.64
18	*NPM1_NRAS*	0.563	0.57	0.64
19	*KRAS_NRAS*	-0.550	0.58	0.64
20	*PTPN11_WT1*	-0.036	0.97	0.98
21	*IDH2_PTPN11*	0.021	0.98	0.98
